# Protein Tyrosine Phosphatase Regulates Sporulation, Trap Morphogenesis, Stress Responses, and Secondary Metabolism in *Arthrobotrys oligospora*

**DOI:** 10.3390/jof12080595

**Published:** 2026-08-11

**Authors:** Guiqiu Luo, Lirong Zhu, Hui Yuan, Lihua Wei, Yi Chen, Xuemei Li, Jinkui Yang

**Affiliations:** State Key Laboratory for Conservation and Utilization of Bio-Resources in Yunnan, Yunnan Key Laboratory of Basic Research and Innovative Application for Green Biological Production, School of Life Sciences, Yunnan University, Kunming 650091, China; luoguiqiu@stu.ynu.edu.cn (G.L.); zhulirong_dif5@stu.ynu.edu.cn (L.Z.); yuanhui201228@163.com (H.Y.); lihuaq202503@163.com (L.W.); 12024214011@stu.ynu.edu.cn (Y.C.); xmli@mail.ynu.edu.cn (X.L.)

**Keywords:** protein tyrosine phosphatase, sporulation, trap formation, stress response

## Abstract

*Arthrobotrys oligospora* is a widely distributed nematode-trapping (NT) fungus that captures nematodes by developing flexible traps. In fungi, protein tyrosine phosphatases (PTPs) are crucial for intracellular signaling, governing processes such as cell growth, proliferation, and differentiation in filamentous species. In this study, we characterized the functions of AoPtp (an orthologous PTP) through gene knockout, phenotypic, multi-omics, and yeast two-hybrid (Y2H) analyses. Inactivation of *Aoptp* caused a marked increase in trap number and nematode predation ability. The Δ*Aoptp* mutants exhibited enhanced mycelial growth on TYGA and TG media, but showed no significant growth difference on PDA medium, together with reduced spore yield, and altered stress responses. In addition, phenotypic and transcriptomic analyses suggested that AoPtp is associated with lipid droplet accumulation and autophagy-related processes. Metabolomic analysis revealed extensive changes in metabolic profiles, including an approximately six-fold reduction in arthrobotrisin abundance. Furthermore, AoPtp interacts with AoFus3 and AoSlt2 in a Y2H assay, suggesting its potential involvement in the mitogen-activated protein kinase signaling pathway. In summary, we demonstrated that AoPtp is a pleiotropic regulator of sporulation, trap development, stress tolerance, and metabolic process in *A. oligospora*. These findings provide a basis for probing the regulatory mechanism of PTPs underlying trap formation in NT fungi, as well as for exploring their potential applications in biocontrol of nematode-associated diseases.

## 1. Introduction

Nematode-trapping (NT) fungi are promising biocontrol fungi that can lure, kill, and digest nematodes through specialized trapping structures (traps), such as adhesive knobs, adhesive networks, and constricting rings [1,2,3]. These traps are indispensable weapons to capture nematodes. *Arthrobotrys oligospora*, a typical NT fungus, produces conidia for asexual growth and develops adhesive three-dimensional networks to capture nematodes. It has been intensively investigated as a model organism to probe the interaction between fungi and nematodes [4]. Multiple protein kinases, such as conserved mitogen-activated protein kinases (MAPKs) [5] and serine/threonine kinases (STKs) [6], are critically involved in vegetative growth and trap development of *A. oligospora*. Phosphorylation and dephosphorylation mediated by kinases and phosphatases, respectively, play vital roles in modulating growth, developmental processes, and extracellular signal transduction in filamentous fungi [7].

Protein phosphorylation is a reversible post-translational modification mechanism that regulates several cellular processes, such as signal transduction, gene expression, and cell division, by covalently adding phosphate groups to protein molecules [8,9,10]. In *Magnaporthe oryzae*, phosphorylation of the G protein-regulated factor MoRgs1 by the tyrosine protein kinase MoCk2 is critical for appressorium development and pathogenicity [11]. Loss of Mka1, an activator of MAPK, results in defects in *M. oryzae*, including reduced aerial hyphal growth, clustering, appressoria formation, and pathogenicity [12]. Additionally, phosphatase-mediated dephosphorylation is indispensable in modulating fungal development and pathogenicity. Disruption of the protein phosphatase MoPtc6 in *M. oryzae* led to a drastic reduction in the development of conidiophore and conidium, impaired hyphal growth, decreased appressorial turgor pressure, and impaired glycogen utilization capacity ultimately resulting in markedly reduced penetration ability and virulence [13]. In *Aspergillus flavus*, the PP2C-type phosphatases Ptc1 and Ptc2 regulate autophagy and aflatoxin biosynthesis by dephosphorylating phosphoglycerate kinase 1 (PGK1); their absence significantly impairs conidial development and virulence [14]. In *Aspergillus fumigatus*, calcineurin-dependent dephosphorylation regulates hyphal morphogenesis, cell wall integrity (CWI), and virulence [15].

Based on differences in substrate specificity, catalytic mechanisms, and sensitivity to inhibitors, eukaryotic protein phosphatases can be classified into four major structural families. The first family comprises serine/threonine-specific protein phosphatases, including PP1, PP2A, and the calcium/calmodulin-regulated PP2B (also known as calmodulin-dependent protein phosphatase) [16,17]. The second family consists of metal-dependent protein phosphatases (PPMs), which also belong to the serine/threonine-specific phosphatases. The third structural family includes protein tyrosine phosphatases (PTPs), which act specifically on phosphorylated tyrosine residues, as well as bispecific phosphatases capable of dephosphorylating all three types of phosphorylated residues. The fourth family includes aspartate-dependent phosphatases [18]. Within the PTP superfamily, phosphorylation levels are bidirectionally regulated by protein tyrosine kinases (PTKs) and PTPs, with the former catalyzing the addition of phosphate groups and the latter mediating their removal [19,20]. PTPs constitute a class of enzymes that act as critical regulators of various biological processes, such as signaling pathways, growth, proliferation, differentiation, transformation, and cellular homeostasis [21,22].

In *Saccharomyces cerevisiae*, Ptp2 and Ptp3 negatively regulate the MAPK signaling pathways including Hog1, Fus3, and Mpk1/Slt2. Furthermore, Ptp2 and Ptp3 are essential for the survival of *S. cerevisiae* under heat stress. The absence of Ptp2 and Ptp3 results in defective sporulation [23,24,25]. In *Cryptococcus neoformans*, Ptp1 and Ptp2 serve as negative regulators of the hyperosmotic glycerol (HOG) pathway [26]. Ptp2 is essential for nutrient-dependent cell growth, sexual development, and the production of virulence factors. *Ptp2* gene knockout disrupts the negative feedback loop in the HOG pathway, differentially affecting environmental stress responses and adaptability [26]. Ptp1, acting as a backup PTP for Ptp2, plays a secondary role in regulating growth, stress tolerance, and virulence in *C. neoformans* [26]. In *Botrytis cinerea*, BcPtpA and BcPtpB positively regulate the phosphorylation levels of BcSak1 (an Hog1 homolog) and BcBmp3 (an Mpk1 homolog) under stress conditions. Furthermore, BcPtpA and BcPtpB play critical regulatory roles in mycelial growth, pathogenicity, and adaptation to multiple chemical stressors in *B. cinerea* [27]. In summary, PTPs have been shown to regulate fungal vegetative growth, stress responses, sexual differentiation, and virulence through the MAPK signaling pathway.

The role of MAPKs (Hog1, Fus3, and Mpk1/Slt2) has been extensively studied in *A. oligospora* [28,29]. In *A. oligospora*, Slt2 functions as a core member of the CWI pathway, and its inactivation abolishes sporulation and trap development [30]. Similarly, disruption of *fus3* resulted in reduced spore yield and trap production, with hyphal fusion completely abolished [31]. However, the functions of PTPs and their potential relationships with MAPK signaling pathways in NT fungi remain largely unknown. Here, we investigated the biological functions of an orthologous PTP AoPtp in *A. oligospora* through gene knockout, phenotypic characterization, multi-omics analyses, and Y2H assays, with the aim of elucidating its potential roles in mycelial development, trap formation, stress adaptation, and MAPK-associated signaling.

## 2. Materials and Methods

### 2.1. Fungal Strains and Culture Conditions

Wild-type (WT) *A. oligospora* (ATCC 24927) (originally obtained from the American Type Culture Collection (ATCC) and maintained in our laboratory) and the Δ*Aoptp* mutant strains were cultivated on potato dextrose agar (PDA) plates at 28 °C. PDA supplemented with sucrose (PDAS) medium was used for protoplast incubation. Mycelial growth rates were determined on PDA, tryptone–yeast extract–glucose agar (TYGA), and tryptone–glucose (TG) plates as previously described [4]. Spore yields were assessed on corn meal–yeast extract (CMY) medium, and spore germination and trap induction experiments were conducted on water agar (WA: agar 20 g/L) medium [32]. The free-living nematode *Caenorhabditis elegans* strain N2 was incubated on oat medium (oats: ddH_2_O = 1:1) at 28 °C. In addition, the *S. cerevisiae* FY834 strain was spread on yeast extract–peptone–dextrose (YPD) plates at 30 °C for the construction of knockout vectors, while *Escherichia coli* DH5α was maintained on Luria–Bertani plates at 37 °C as the host for the plasmids pRS426 and pCSN44 [33]. Unless otherwise indicated, all media were prepared in our laboratory using analytical-grade reagents purchased from Tianjin Hengxing Chemical Reagent Manufacturing Co., Ltd. (Tianjin, China).

### 2.2. Sequence Analysis of AoPtp

The protein sequence of AoPtp (AOL_s00169g50) was searched from *A. oligospora*, and homologs from various fungi were retrieved by Protein BLAST alignment (https://blast.ncbi.nlm.nih.gov/, accessed on 20 March 2026). The similarity between AoPtp and its homologs was predicted using DNAman software (version 60399), and conserved domains were identified using InterProScan (https://www.ebi.ac.uk/interpro/, accessed on 20 March 2026), and then visualized with Tbtools-II (2.3.3.0) [5]. In addition, the physicochemical properties of AoPtp were predicted using the pI/MW software (https://web.expasy.org/compute_pi/, accessed on 20 March 2026) [5]. A phylogenetic tree was constructed using the MEGA 11 software [33].

### 2.3. Gene Knockout of Aoptp

The sequence encoding *Aoptp*, along with 2000 bp of upstream and downstream regions of the gene, was searched from the *A. oligospora* genome. The 5′ and 3′ flanking fragments of *Aoptp* and the *hph* cassette were amplified using paired primers (Table S1), then co-transformed with linearized pRS426 into *S. cerevisiae* FY834 for in vivo homologous recombination. The assembled disruption cassette was subsequently amplified using specific primers (Table S1) and transformed into *A. oligospora* protoplasts [33]. The protoplasts were spread on PDAS plates containing 200 μg/mL hygromycin B (Amresco, Solon, OH, USA) at 28 °C and cultured for a week [29]. Colonies were picked, cultured on TYGA plates, and confirmed using PCR and real-time quantitative PCR (RT-qPCR) methods [29].

### 2.4. Analyses of Phenotypic Traits

Fresh colony discs of WT and Δ*Aoptp* mutant strains were cultivated on PDA, TYGA, and TG plates, and the colony diameters were measured at 24 h intervals. To determine the spore yield, fungal strains were cultivated in CMY medium for ten days. Deionized water (15 mL) and an appropriate amount of glass beads were added to dislodge the conidia from the colony surface. The mixture was filtered and passed through a funnel to obtain a spore suspension. To calculate the spore germination rates, 20,000 spores were incubated on WA plate and observed under a light microscope at 3–9 h [31].

To induce the trap formation of the fungal strains, 5 μL of spore suspension (2 × 10^4^ spores) was spread on WA medium and cultured for 2–3 days. Approximately 300–400 *C. elegans* N2 nematodes were added to induce trap formation. The trap morphology and captured nematodes were observed, and the trap numbers and nematocidal activity were calculated at 12–48 h post-induction (hpi) [34]. Each newly formed and spatially distinct trap of every WA plate was counted as one independent trap, irrespective of the number of hyphal loops within the trap. The total number of independent traps formed on each plate was counted at the indicated time points for the WT strain and three Δ*Aoptp* mutants. Three replicates were performed for each strain.

### 2.5. Analyses of Stress Response

To analyze the role of AoPtp in stress tolerance, fresh mycelial discs from WT and Δ*Aoptp* mutant strains were inoculated on TG plates supplemented with menadione (0.05, 0.07 and 0.09 mM), H_2_O_2_ (5, 10, and 15 mM), Congo red 0.10 and 0.14 mg/mL, SDS (0.01%, 0.02%, and 0.03%), NaCl (0.10, 0.20 and 0.30 M), or sorbitol (0.25, 0.50 and 0.75 M). These concentrations were selected based on previous studies [35]. Colony diameters were measured after five days of incubation. Relative growth inhibition was calculated as RGI (%) = [(Dc − Dt)/Dc] × 100, where Dc and Dt represent the colony diameters of the same strain on control and stressor-containing TG medium, respectively [5].

### 2.6. Fluorescent Staining

Fresh mycelia were cultivated on PDA plates covered with cover slips for five days. Mycelia growing on the cover slips were treated with 20 μg/mL calcofluor white (CFW, Sigma-Aldrich, St. Louis, MO, USA) and 20 μg/mL 4′, 6-diamidino-2-phenylindole (DAPI, Sigma-Aldrich) to visualize the cell septa and nuclei. Similarly, 10 µg/mL boron dipyrromethene (BODIPY, Sigma-Aldrich) was used to stain lipid droplets (LDs) and 100 µg/mL mono dansyl cadaverine (MDC, Sigma-Aldrich) was used to visualize MDC-positive autophagy-related structures. Stained mycelia were observed using a Nikon fluorescence microscope [36].

### 2.7. RT-qPCR Analysis

RNA samples were isolated from WT and Δ*Aoptp* mutant strains using Trizol™ reagent (UElandy, Suzhou, China), followed by reverse transcription using the PrimeScript RT Kit (Takara, Dalian, China) according to the manufacturer’s instructions. Primers used for RT-qPCR analysis are listed in Tables S1 and S2. β-tubulin was selected as the internal reference because its expression remained stable in our experimental conditions, and the expression of each gene was detected using the 2^−ΔΔCT^ method [37].

### 2.8. Transcriptome Analysis

Fresh mycelia discs were cultivated in liquid TG medium at 28 °C at 180 rpm for two days [4]. The mycelia were separated using a funnel and spread on WA plates. One thousand nematodes were added to each plate (three replicates/sample) to induce trap formation. The mycelial samples were harvested at 0 and 24 hpi and sent to Guangzhou Gideao Technology Service Co., Ltd. for RNA sequencing [5]. Transcriptome data were processed using the OmicSmart cloud platform (https://www.omicsmart.com/, accessed on 18 April 2026).

### 2.9. Metabolomic Analysis

Equal amounts of mycelial sample from the WT and mutant strains were cultivated in PD broth at 28 °C at 180 rpm for seven days. The mycelial samples and supernatants were separated using a funnel, and then an equal volume of ethyl acetate was added to the supernatant. The mixture was mixed thoroughly by shaking and then allowed to stand for 12 h. The supernatant was rotary-evaporated, resuspended in 1–2 mL of methanol, and transferred to a brown vial. After the methanol had completely evaporated, the solution was quantified based on the weight of the mycelia for liquid chromatography–mass spectrophotometric (LC-MS) analysis (Thermo Fisher Scientific, Bremen, Germany). Subsequently, the metabolites were identified and analyzed using the Compound Discoverer 3.0 software package [29]. Differential metabolites were identified based on fold change and statistical significance. Metabolites with fold change ≥ 2 and *p*-value < 0.05 were considered significantly differentially abundant metabolites.

### 2.10. AoPtp Interacting Proteins and Y2H Analysis

Using the STRING database, we predicted the potential AoPtp interacting proteins. Primers were designed using the Vazyme primer design tool (URL: https://crm.vazyme.com/cetool/simple.html, accessed on 20 May 2026). The genes encoding AoPtp and its interacting proteins were amplified using these paired primers (Table S3). The *Aoptp* coding sequence was cloned into the pGBKT7 plasmid (MedChemExpress, New Jersey, NJ, USA) as the bait construct. The purified coding sequences of AoLsg1, AoPkd1, AoSs1g, AoCnaA, AoPp1, AoMid1, AoFus3, AoHog1, and AoSlt2 were cloned into pGADT7 vector as prey constructs. The pGADT7-target plasmids along with the pGBKT7-Ptp plasmid were co-transfected into the Y2HGold yeast receptor cells according to the manufacturer’s instructions (Weidi, Shanghai, China). Transformants were initially selected on SD/−Leu/−Trp medium (Coolaber, Beijing, China) and subsequently cultured on SD/−Leu/−Trp/−His/−Ade medium (Coolaber) supplemented with X-α-Gal (Weidi) to assess protein interactions. The pGBKT7-53/pGADT7 and pGBKT7-Lam/pGADT7 combinations were used as positive and negative controls, respectively. Autoactivation was evaluated by co-transforming the bait plasmid with empty pGADT7, and each prey plasmid with empty pGBKT7. The Y2H assay was carried out as described previously [6,32].

### 2.11. Statistical Analyses

Experimental data are expressed as the mean ± standard deviation of three repetitions. Statistical analysis was performed using Prism 8.0 (GraphPad Software). The variance between the WT strain and mutant was assessed using Tukey’s honestly significant difference (HSD) test. Statistical significance was set at *p* < 0.05.

## 3. Results

### 3.1. Sequence Analysis and Aoptp Knockout

AoPtp encodes a protein containing 958 amino acid residues, with an isoelectric point of 9.61 and a molecular weight of 103.26 kDa. Cluster analysis indicated that AoPtp homologs from NT and other filamentous fungi form two distinct branches (Figure S1A). Sequence alignment results showed that AoPtp shares high similarity (78.52–95.72%) with homologous proteins from other NT fungi, with the highest similarity (95.72%) with *Arthrobotrys flagrans* (syn. *Duddingtonia flagrans*). In contrast, similarity of AoPtp to homologs from other fungi ranged from 33.47% to 38.11%. Structural domain analysis revealed that AoPtp homologs contain a conserved dual-specificity phosphatase (DSP) fungal SDP1-like domain. AoPtp belongs to the PTP_DSP_cys superfamily, which comprises cysteine-based phosphatases, including classical PTPs as well as DUSPs or DSPs. To probe the role of *Aoptp*, we disrupted the *Aoptp* gene in *A. oligospora* via homologous recombination. Three independent transformants were successfully obtained, verified through PCR (Figure S1B,C) and RT-qPCR (Figure S1D), and used for downstream experiments.

### 3.2. AoPtp Regulates Mycelial Growth and Nuclear Distribution

**Figure 1:**
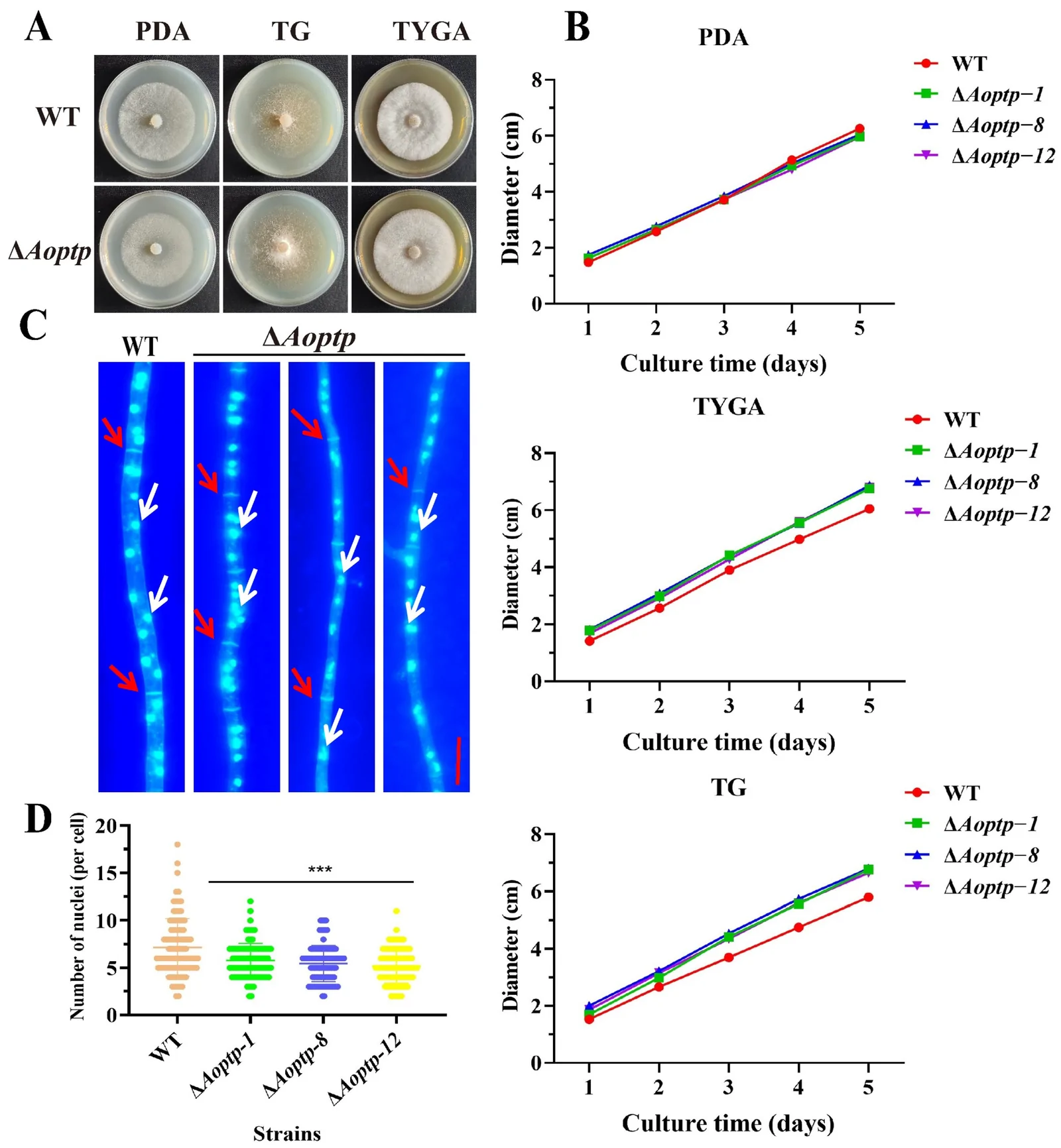
Comparison of mycelial growth and development between the wild-type (WT) and protein tyrosine phosphatase mutant (Δ*Aoptp*) strains of *A. oligospora*. (**A**) Colonies of WT and Δ*Aoptp* mutant strains were incubated on different media for five days. (**B**) Mycelial growth rates on PDA, TYGA, and TG plates. (**C**) Comparison of hyphal septa and nuclei. Bar = 10 μm. Red arrows indicate hyphal septa, and white arrows indicate nuclei. (**D**) The number of nuclei per hyphal cell. Significant differences between the Δ*Aoptp* mutants and the WT strain are indicated with asterisks (values represent mean ± SD, *n* = 3; Tukey’s HSD test, *** *p* < 0.001).

Compared with the WT strain, the Δ*Aoptp* mutants exhibited medium-dependent growth alterations, with enhanced growth on TYGA and TG media but no significant difference on PDA medium. Although the Δ*Aoptp* mutant strains showed no significant difference in growth on PDA plates, they grew faster on TYGA and TG plates than the WT (Figure 1A,B). The hyphae were stained with CFW and DAPI to visualize hyphal septa and nuclei, respectively, and 90 samples were randomly quantified for each strain. The hyphal cell length of the Δ*Aoptp* mutant was comparable to that of the WT (Figure S2A,B). In contrast, the Δ*Aoptp* significantly reduced the nuclear number in hyphal cells, as the mutant contained an average of approximately five nuclei per hyphal cell, whereas the WT strain contained approximately eight (Figure 1C,D).

### 3.3. AoPtp Regulates Spore Production and Spore Germination

**Figure 2:**
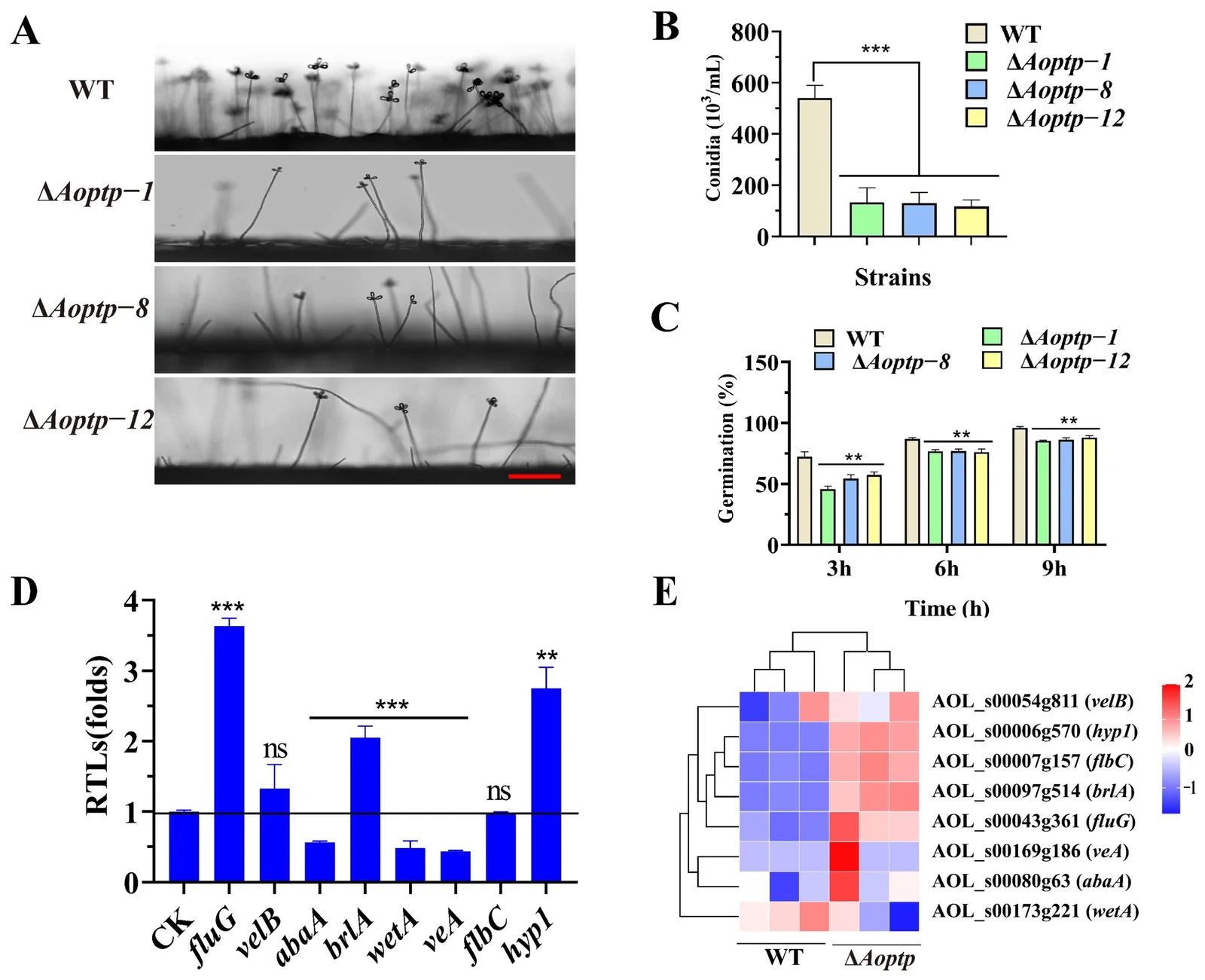
Comparison of conidiophores and spore yields between the WT and Δ*Aoptp* mutant strains. (**A**) Observation of the conidiophores. Bar = 50 μm. (**B**) Quantification of spore yields. (**C**) Spore germination rates. (**D**) Relative transcription levels (RTLs) of sporulation-related genes. (**E**) Heatmap of transcriptional levels of sporulation-related genes. Significant differences between the Δ*Aoptp* mutants and the WT strain are indicated with asterisks (data represent mean ± SD, *n* = 3; Tukey’s HSD test, ** *p* < 0.01; *** *p* < 0.001; ns, no significant difference).

Morphology of the conidiophores from Δ*Aoptp* mutants showed no significant difference relative to the WT (Figure 2A). After ten days of incubation on CMY medium, the spores were collected and quantified. The WT strain produced 5 × 10^5^ conidia/mL, whereas the Δ*Aoptp* mutants produced 1 × 10^5^ conidia/mL (Figure 2B). In addition, compared to WT, the germination rates of Δ*Aoptp* spores at 3, 6, and 9 h were markedly reduced (Figure 2C). Next, we measured the transcription of sporulation-related genes by RT-qPCR, and found that the expression levels of the core sporulation genes *abaA*, *wetA* and *veA* were remarkably reduced, whereas those of *fluG*, *brlA*, and *hyp1* were remarkably increased (Figure 2D). In addition, transcriptome data showed that the transcription patterns of these genes were similar to the RT-qPCR analysis (Figure 2E).

### 3.4. AoPtp Modulates Trap Formation and Hyphal Fusion

**Figure 3:**
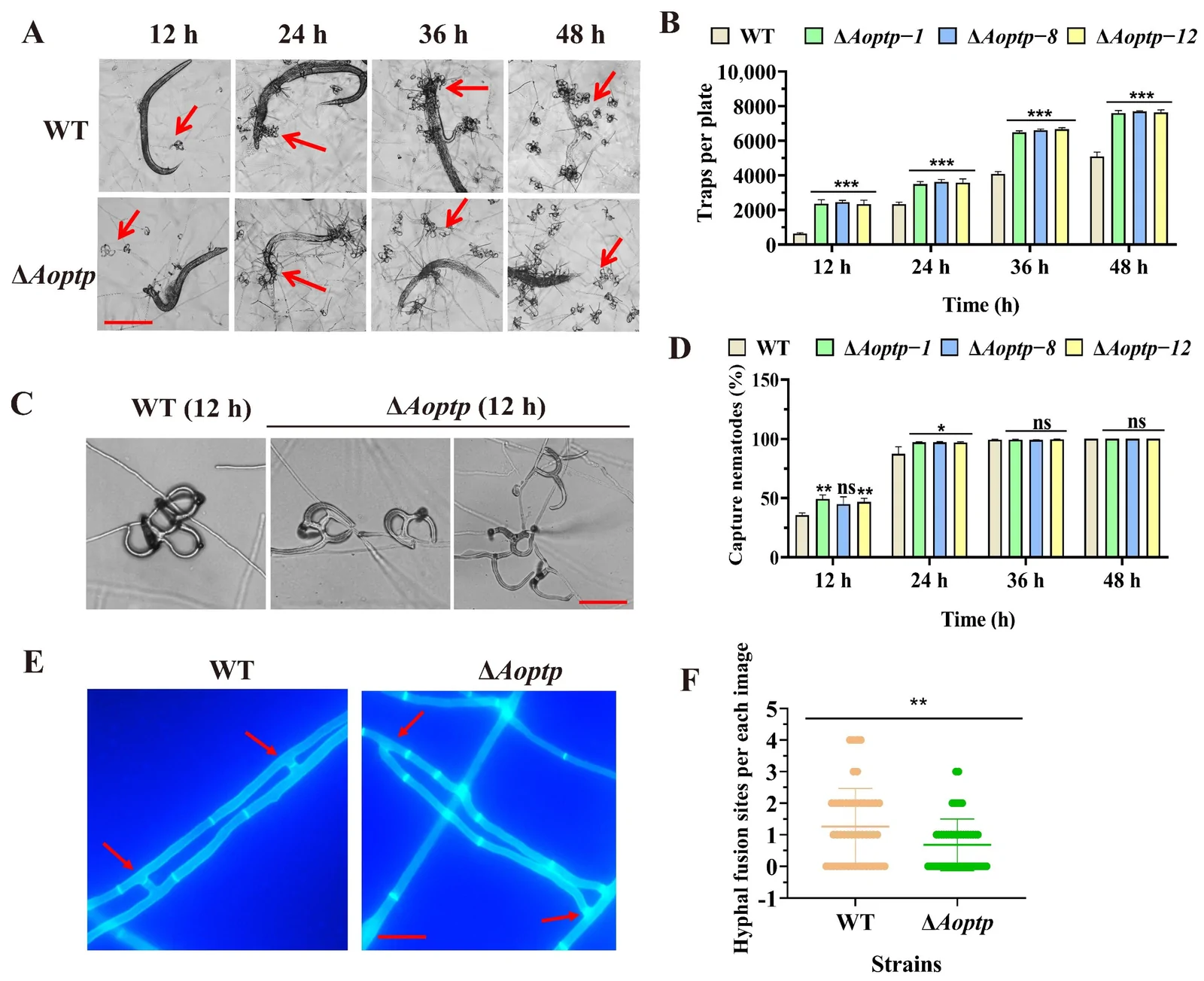
Comparison of trap formation, nematode predation ability, and hyphal fusion. (**A**) Representative images of trap formation and captured nematodes. The red arrow points to the trap. Bar = 100 μm. (**B**) Quantification of trap numbers in the WT and Δ*Aoptp* mutant strains. Each spatially distinct trap was counted as one trap. Data are presented as the mean ± SD from three independent biological replicates for the WT strain and Δ*Aoptp* mutants. (**C**) Morphology of traps at 12 h. Bar = 20 μm. (**D**) Nematode predation ability of the WT and Δ*Aoptp* mutant strains. (**E**) Observation of the hyphal fusion bridges. The red arrow points to the hyphal fusion sites. Bar = 10 μm. (**F**) Comparison of hyphal fusion sites (50 images were compared). Significant differences between the Δ*Aoptp* mutants and the WT strain are indicated with asterisks (data represent mean ± SD, *n* = 3; Tukey’s HSD test, * *p* < 0.05; ** *p* < 0.01; *** *p* < 0.001; ns, no significant difference).

To analyze the role of AoPtp in trap development and nematode predation, the traps were quantified after induction with nematodes. At tested time points, the trap production was remarkably increased in the Δ*Aoptp* mutant strains relative to the WT strain (Figure 3A–C). Furthermore, the predation ability of the Δ*Aoptp* mutant strains was higher than that of the WT strain at 12 h and 24 h. The nematode mortality rate in both strains reached 100% by 36 hpi and 48 hpi (Figure 3D). Moreover, observation of the trap morphology revealed that the WT traps consisted of more hyphal loops than the Δ*Aoptp* mutant strains at 12 hpi (Figure 3C). Furthermore, *Aoptp* inactivation caused a marked reduction in hyphal fusion bridges (Figure 3E,F).

### 3.5. AoPtp Modulates Multiple Stress Responses

**Figure 4:**
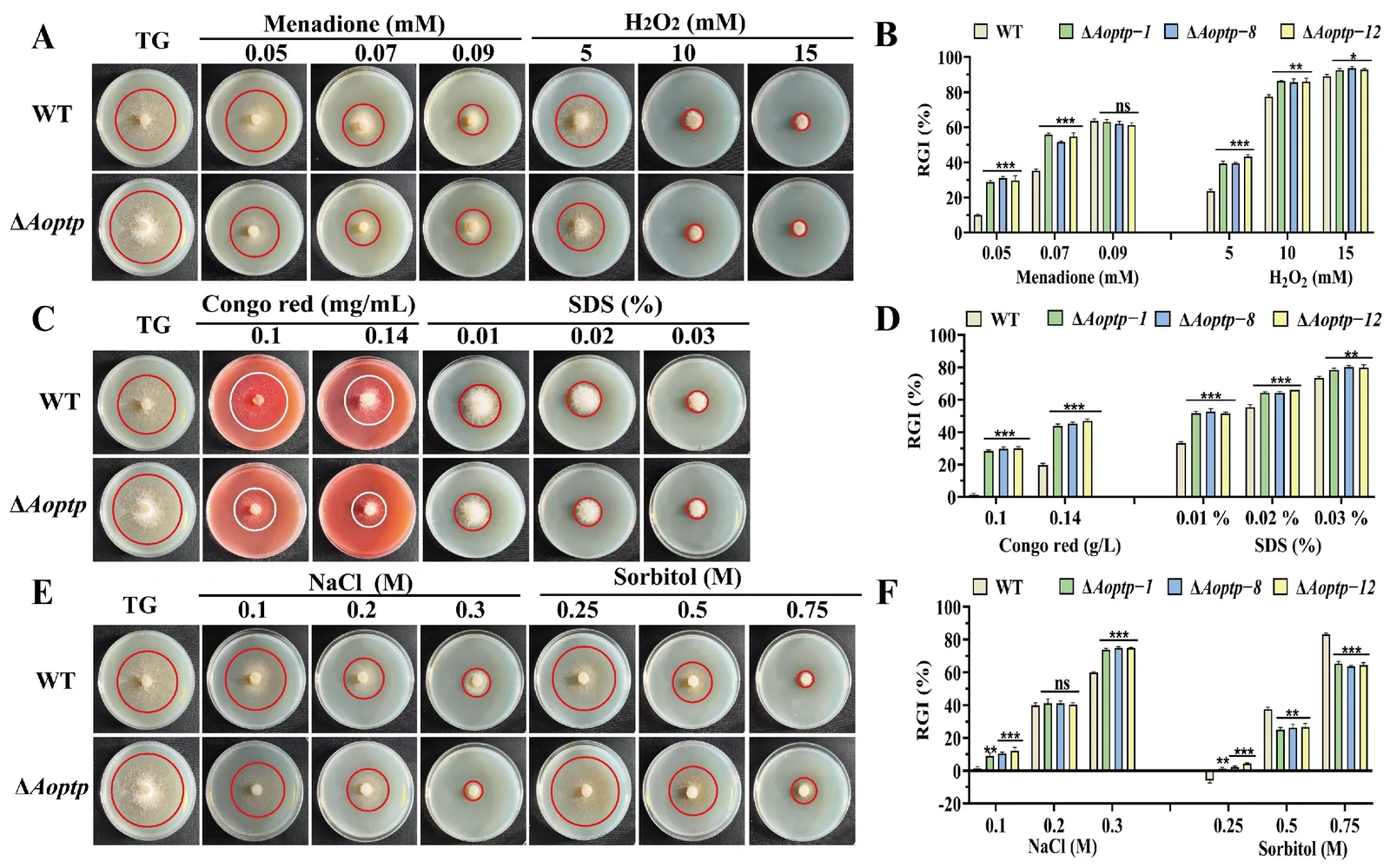
Comparison of stress response to various chemical reagents. (**A**,**C**,**E**) Colonies of the WT and Δ*Aoptp* mutant strains incubated on TG media containing various stressors, including oxidative reagents (**A**), cell-wall-disturbing reagents (**C**), and osmotic reagents (**E**). The red circle represents the colony size. (**B**,**D**,**F**) Relative growth inhibition (RGI) values under various stressors, including oxidative reagents (**B**), cell-wall-disturbing reagents (**D**), and osmotic reagents (**F**). Significant differences between the Δ*Aoptp* mutants and the WT strain are indicated with asterisks (data represent mean ± SD, *n* = 3; Tukey’s HSD test, * *p* < 0.05; ** *p* < 0.01; *** *p* < 0.001; ns, no significant difference).

Fungal strains cultivated on TG plates containing various chemical reagents for five days exhibited varying degrees of mycelial growth inhibition. The Δ*Aoptp* mutant strains were more susceptive to the oxidative reagents menadione and H_2_O_2_, and exhibited higher RGI values than WT at 0.05 mM and 0.07 mM menadione and at 5–15 mM H_2_O_2_ (Figure 4A,B). Similarly, the Δ*Aoptp* mutant strain exhibited markedly higher RGI values than the WT strain when treated with the cell-wall-disturbing reagents Congo red (0.1–0.14 mg/mL) and SDS (0.01–0.03%) (Figure 4C,D). However, the Δ*Aoptp* mutant strains showed different stress tolerance to the osmotic reagents NaCl and sorbitol, exhibiting markedly increased RGI values when treated with 0.1 and 0.3 M NaCl, and 0.25 M sorbitol, but reduced RGI values with 0.5 and 0.75 M sorbitol (Figure 4E,F).

### 3.6. AoPtp Inactivation Affects Gene Transcription

**Figure 5:**
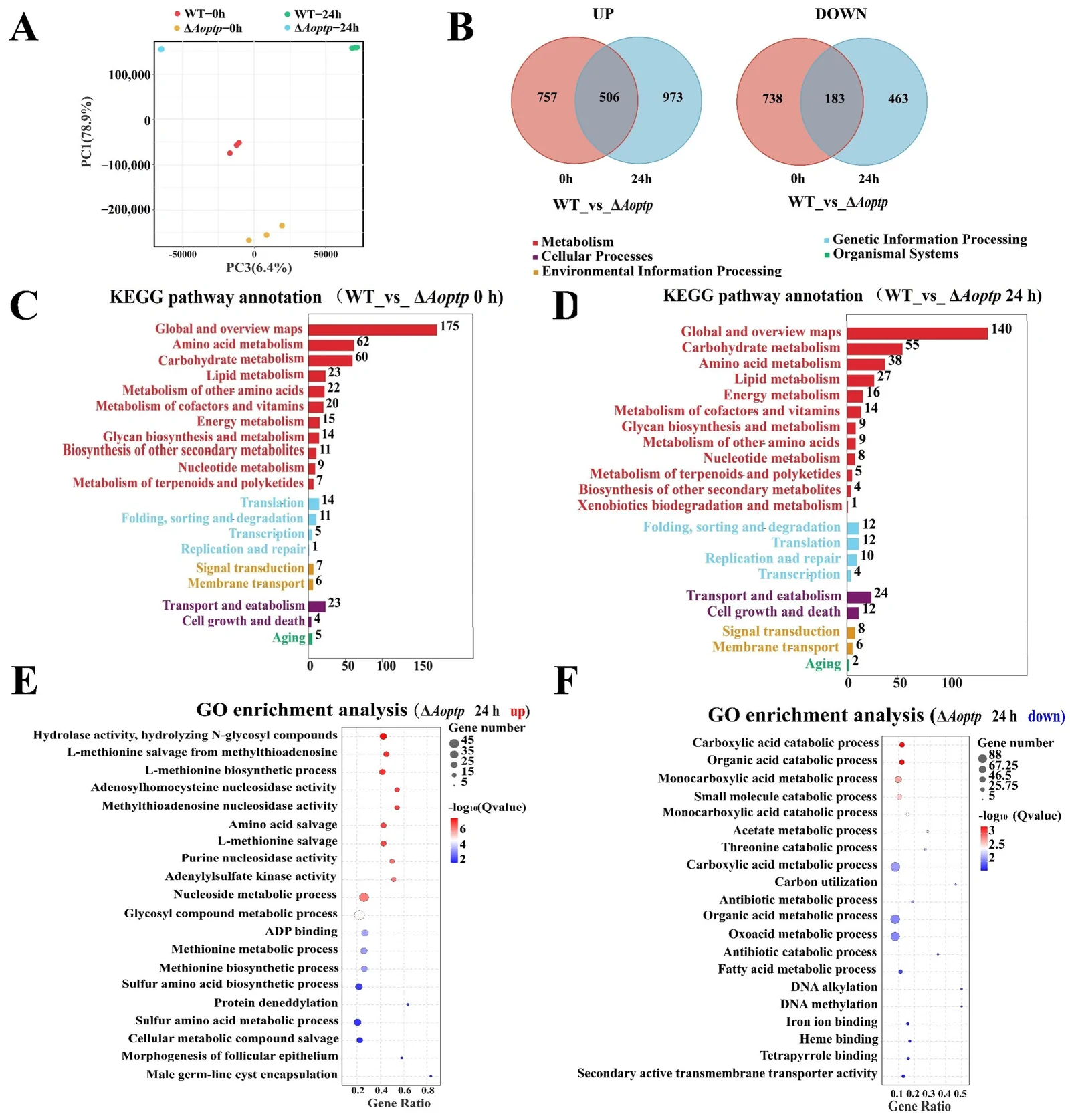
Analysis of transcriptomic data from WT and Δ*Aoptp* mutant strains. (**A**) Principal component analysis (PCA) plots at 0 h and 24 h. (**B**) Venn diagram of upregulated and downregulated genes at 0 and 24 h. (**C**,**D**) KEGG enrichment analysis of DEGs in the Δ*Aoptp* mutant versus WT strains at 0 h (**C**) and 24 h (**D**). (**E**,**F**) GO enrichment analysis of the top 20 upregulated genes (**E**) and downregulated genes (**F**) in the Δ*Aoptp* mutant versus WT strains at 24 h.

We used RNA sequencing to determine the whole-genome transcriptional levels in the WT and Δ*Aoptp* mutant strains under both no-nematode-induction (0 h) and nematode-induction conditions (24 h). Principal component analysis revealed a clustered distribution among samples within the same group (Figure 5A). Venn diagram analysis revealed 1263 upregulated and 921 downregulated differentially expressed genes (DEGs) in the Δ*Aoptp* mutant relative to the WT at 0 h, whereas 1479 upregulated and 646 downregulated DEGs were found in the mutant strain at 24 h. Venn diagram analysis showed that 506 upregulated and 183 downregulated DEGs were collectively enriched at 0 h and 24 h (Figure 5B), suggesting that gene expression levels undergo significant changes during trap formation in *A. oligospora*.

Subsequently, we performed KEGG (Kyoto Encyclopedia of Genes and Genomes) enrichment analysis on these DEGs at 0 h and 24 h. Multiple metabolism-related processes, such as amino acid, lipid, and carbohydrate metabolism, DNA replication and repair, and cell growth and death were significantly co-enriched at the two time points (Figure 5C,D), suggesting that *A. oligospora* undergoes dynamic growth and development during trap formation. When *A. oligospora* receives a signal from the nematodes, it initiates DNA replication and mobilizes all its energy and resources (particularly lipids, carbohydrates, and amino acids) to produce traps to capture and digest the nematode.

Similarly, GO enrichment analysis of these DEGs was carried out. When comparing 0 h and 24 h, the top 20 GO terms ranked by enrichment level were visualized as a bubble plot. At 0 h, the upregulated DEGs were enriched primarily in terms such as extracellular region, component of plasma membrane, amino acid metabolism, antioxidant activity, and hydrolase activity (Figure S3A), whereas downregulated DEGs were primarily enriched in terms such as oxidoreductase, organic acid metabolic/biosynthetic, carboxylic acid metabolic/biosynthetic, and amino acid metabolic, and small molecular biosynthetic processes (Figure S3B). At 24 h, the upregulated DEGs were primarily enriched in terms such as hydrolase activity, L-methionine biosynthetic/metabolic processes, adenosylhomocysteine nucleosidase activity, and amino acid salvage (Figure 5E). In contrast, the downregulated genes were primarily involved in various catabolic processes, such as carboxylic acid, organic acid, small molecule, and fatty acid (Figure 5F). These data suggest that the absence of *Aoptp* affects global gene transcription levels during vegetative growth and trap development processes.

### 3.7. AoPtp Affects Lipid Metabolism and Autophagy

**Figure 6:**
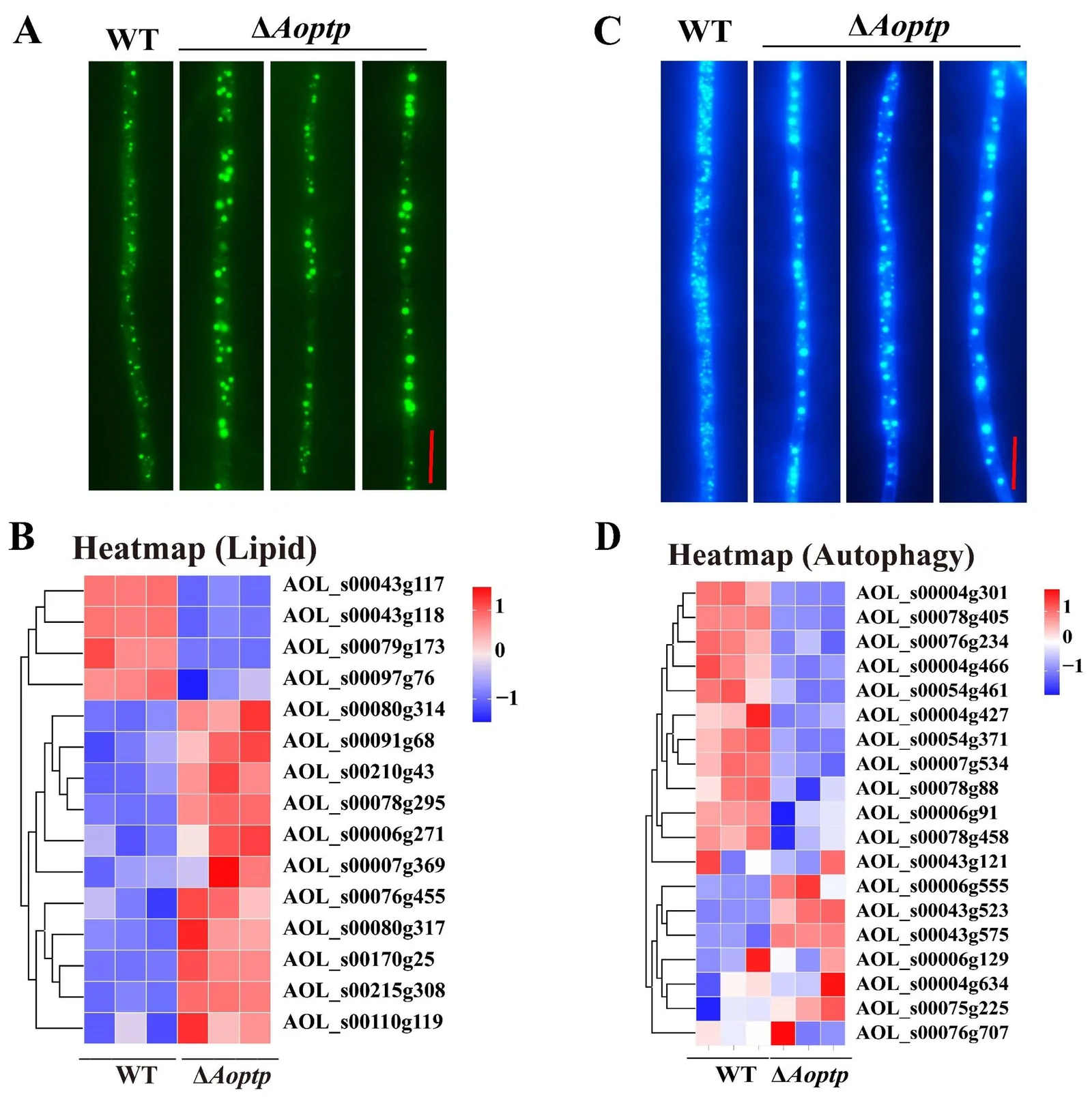
Comparison of lipid metabolism and autophagy. (**A**) Lipid droplets were visualized using boron dipyrromethene (BODIPY) staining, bar = 10 μm. (**B**) Clustering heatmap of lipid metabolism-related genes from the transcriptomic analysis. (**C**) Autophagosomes were visualized using mono dansyl cadaverine (MDC) staining, bar = 10 μm. (**D**) Clustering heatmap of autophagy-related genes from the transcriptomic analysis.

Transcriptome data indicated that several DEGs were involved in lipid metabolism. To verify the roles of AoPtp in lipid metabolism, the mycelia were stained with BODIPY dye. LD volume in the Δ*Aoptp* mutant was markedly greater than that in the WT (Figure 6A). Consistent with this, the transcription of lipid metabolism-related genes was generally upregulated (Figure 6B). Similarly, to determine the role of AoPtp in autophagic process, fresh mycelia were treated with MDC dye. The number of autophagosomes in the Δ*Aoptp* mutant was lower relative to that in the WT, whereas the volume of autophagosomes in the Δ*Aoptp* mutant was higher (Figure 6C). Clustering analysis showed that more autophagy-related genes were downregulated in the Δ*Aoptp* mutant strain (Figure 6D).

### 3.8. AoPtp Is Involved in Secondary Metabolism

**Figure 7:**
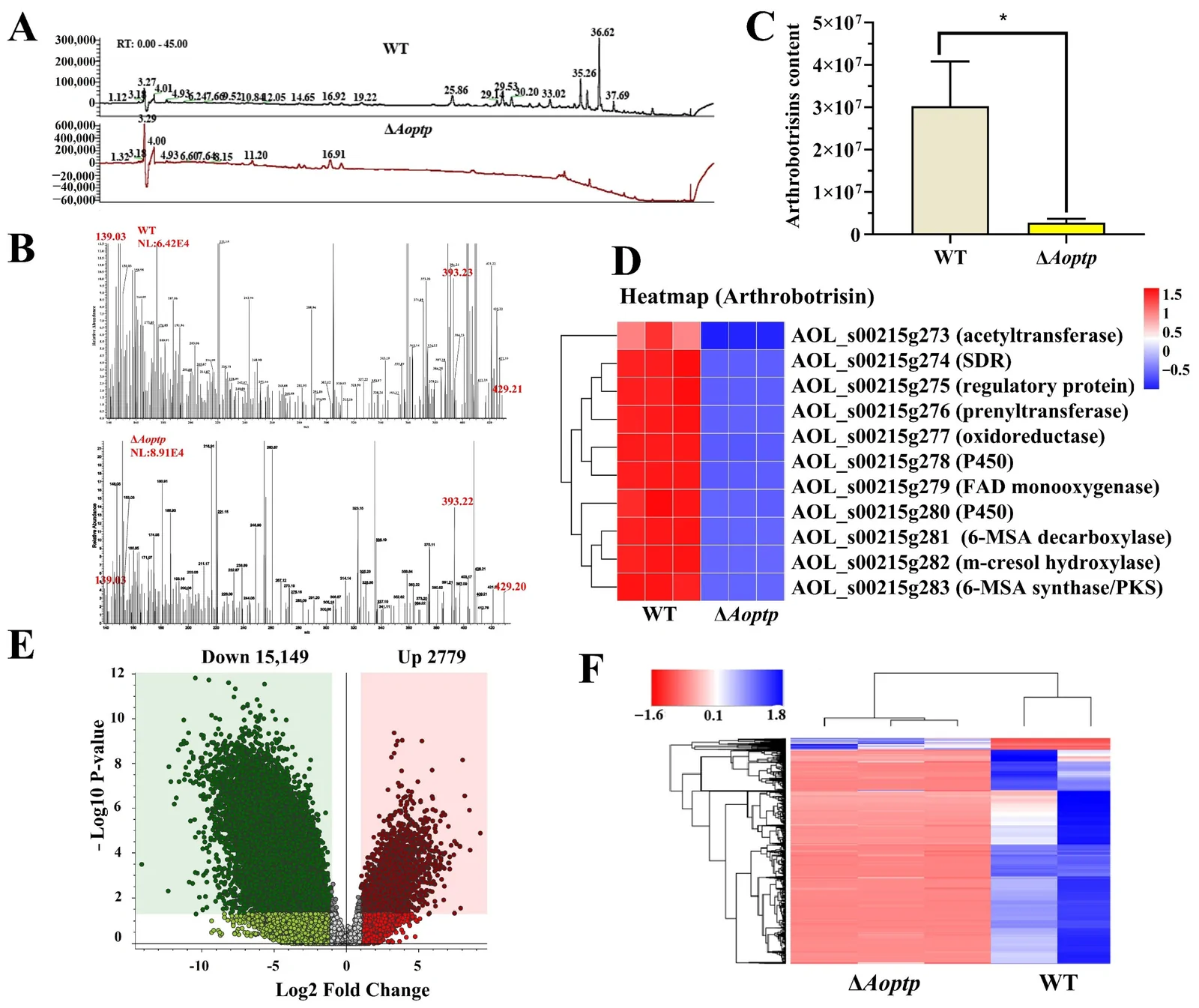
Comparison of secondary metabolites between WT and Δ*Aoptp* mutant strains. (**A**) Comparison of high-performance liquid chromatography (HPLC) profiles. (**B**) Identification of arthrobotrisin based on diagnostic ion fragments at *m*/*z* 139, 393, and 429 in the mass spectrum. (**C**) Comparison of arthrobotrisin content. (**D**) Heatmap showing the expression profiles of genes in the AOL_s00215g cluster associated with arthrobotrisin biosynthesis. Predicted functions of the encoded proteins are indicated in parentheses, and detailed annotations are provided in Table S4. (**E**) Volcano plot analysis of differentially expressed metabolites (fold change ≥ 2 and *p*-value < 0.05). (**F**) Heatmap of upregulated and downregulated metabolic pathways. Significant differences between the Δ*Aoptp* mutant and the WT strain are indicated with an asterisk (* *p* < 0.05).

As few metabolic pathways were markedly enriched in the transcriptome analysis, we sought to verify these by metabolomic analysis. High-performance liquid chromatography–mass spectrometry (HPLC-MS) analysis revealed that metabolite abundance was remarkably reduced in the Δ*Aoptp* mutant strain (Figure 7A). Arthrobotrisins, secondary metabolites produced by *A. oligospora* and related NT fungi, were found at a retention time of 35.01 min (Figure 7B). Their abundance was markedly reduced in the Δ*Aoptp* mutant (Figure 7C). Arthrobotrisins are biosynthesized via the 215g gene cluster (Table S4) [36]. Transcriptomic analysis revealed that transcriptional levels of the 215g cluster were markedly downregulated in the mutants (Figure 7D). The differentially expressed metabolites were further analyzed using cluster analysis. Based on the predefined criteria for differential metabolite identification, a total of 17,928 metabolites showed significant changes between WT and Δ*Aoptp* mutants, including 15,149 downregulated and 2779 upregulated metabolites (Figure 7E). Correspondingly, several metabolic pathways were downregulated in the Δ*Aoptp* mutant (Figure 7F).

### 3.9. AoPtp Can Interact with MAPKs AoFus3 and AoSlt2

**Figure 8:**
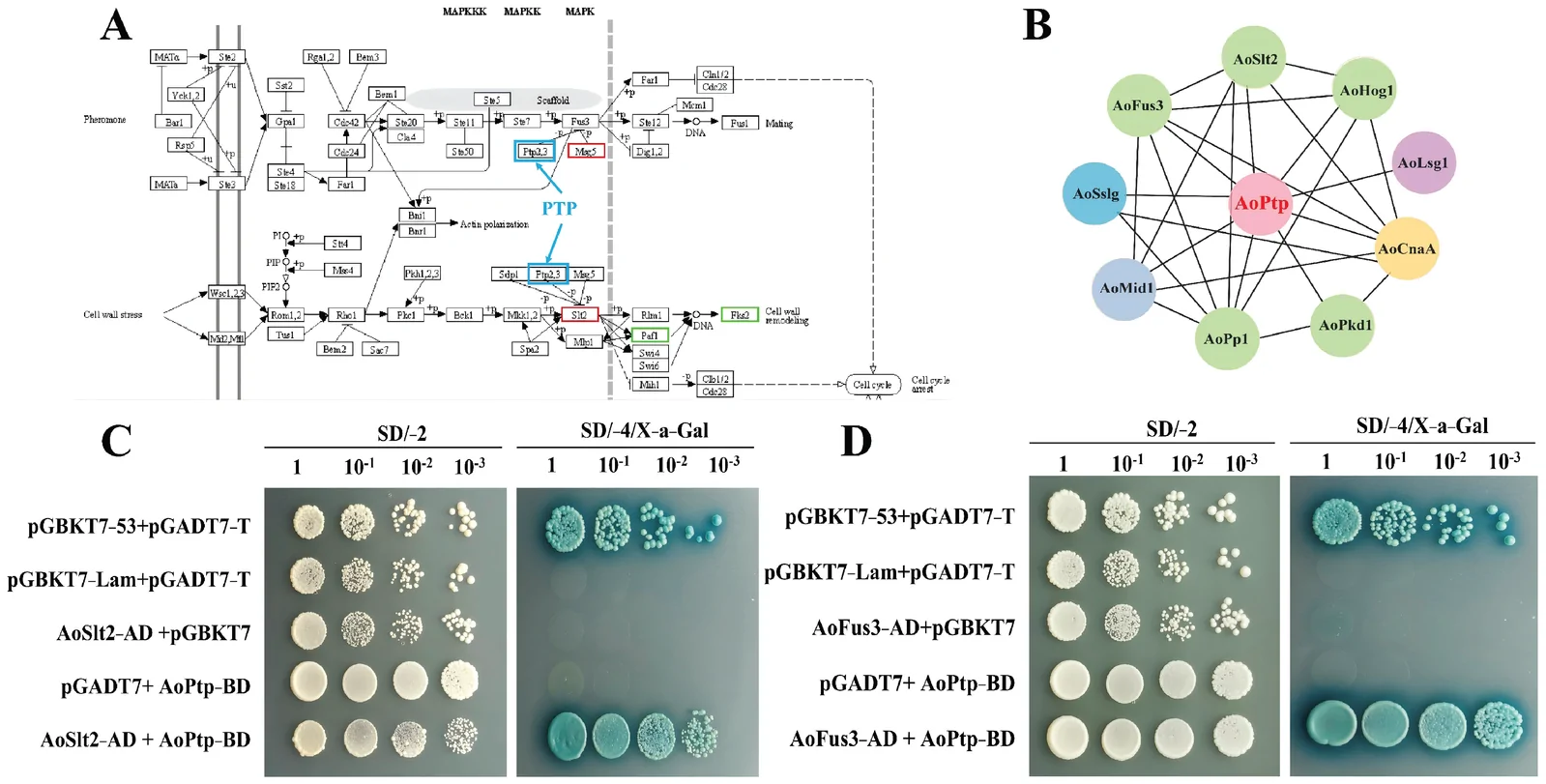
Prediction and identification of AoPtp-interacting proteins. (**A**) Predicted AoPtp-interacting networks based on the KEGG pathway analysis. Dashed lines point PTP, and the colored proteins represent the MAPK-related proteins. (**B**) Predicted AoPtp-interacting networks using STRING database. (**C**,**D**) Verification of the interaction between AoPtp and AoSlt2 (**C**)/AoPtp and AoFus3 (**D**) using yeast two-hybrid assay.

To further investigate the roles and regulatory mechanisms of AoPtp, we predicted AoPtp-interacting proteins using KEGG and STRING database analyses (Figure 8A,B). Nine candidate AoPtp-interacting proteins were predicted: AoLsg1 (AOL_s00079g274), AoPkd1 (AOL_s00054g978), AoSs1g (AOL_s00076g188), AoCnaA (AOL_s00054g224), AoMid1 (AOL_s00079g359), AoPp1 (AOL_s00083g428), AoHog1 (AOL_s00109g23), AoSlt2 (AOL_s00173g235), and AoFus3 (AOL_s00110g154). Y2H validation confirmed that AoFus3 and AoSlt2 interact with AoPtp (Figure 8C,D).

## 4. Discussion

**Figure 9:**
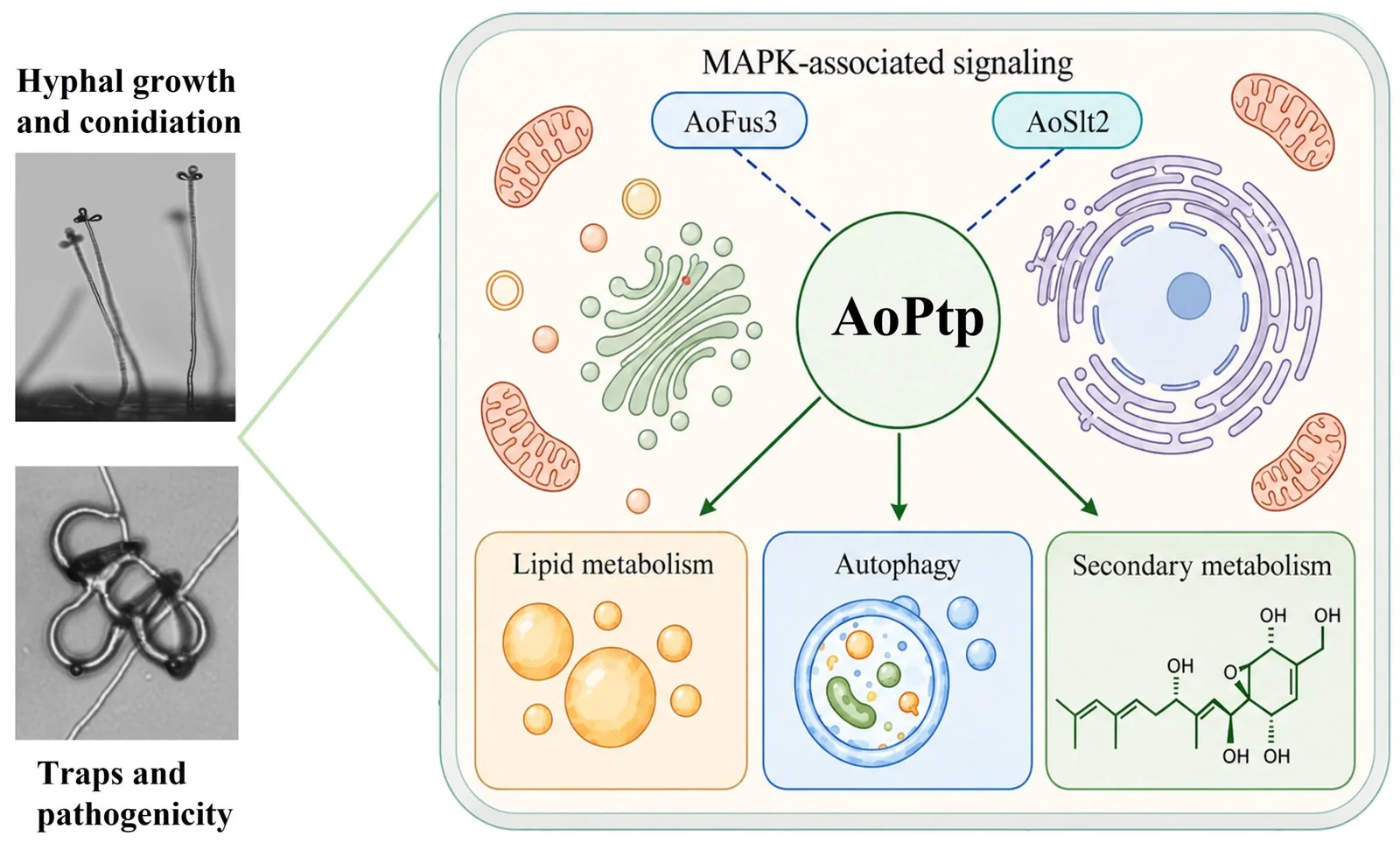
A proposed schematic diagram of the AoPtp regulating sporulation, trap formation and multiple cellular processes in *A. oligospora*.

Protein phosphatases play multiple roles in regulating growth, development, and stress responses by catalyzing dephosphorylation, and they also function as virulence factors in fungi [38]. Here, we systematically analyzed the roles of the protein phosphatase AoPtp in *A. oligospora*. The results indicated that AoPtp is involved in multiple biological processes, including vegetative growth, stress response, asexual reproduction, trap formation, nematode predation, hyphal fusion, nuclear distribution, LD accumulation, and autophagy-related processes (Figure 9).

Relative to the WT, the Δ*Aoptp* mutants exhibited increased growth rates on TG and TYGA media, but no significant difference on PDA medium. These results show that AoPtp is not essential for maintaining basal vegetative growth, but may influence colony expansion under specific nutritional conditions or metabolic states. Similarly, in *Aspergillus cristatus*, the absence of *Acptp2,3* led to altered responses to spore formation and to oxidative, osmotic, and cell wall stress [39]. Previous studies have revealed that PTP-like proteins exert a relatively conserved regulatory role in vegetative growth and environmental adaptation in filamentous fungi. The Δ*Aoptp* mutant exhibited markedly reduced spore production and germination, indicating that Δ*Aoptp* exerts a positive regulatory effect on asexual reproduction and early spore development in *A. oligospora*. Studies in *B. cinerea* have shown that PTP-like proteins influence conidiation; however, the regulatory effects of individual PTP members on spore production are not entirely consistent. These findings suggest that PTP-mediated developmental regulation exhibits both species- and member-specificity [27]. In this study, disruption of *Aoptp* caused a marked change in the transcription of several sporulation-related genes, such as *fluG*, *brlA*, *abaA*, and *wetA*. As an upstream initiator of sporulation, FluG is responsible for initiating the sporulation process and functions as a key regulator of spore formation, while *brlA*, *abaA*, and *wetA* function as central regulatory genes in spore production. In *A. oligospora*, they have been confirmed to be involved in conidiation, trap formation, and predation ability [4]. These results demonstrate that AoPtp regulates conidiation in *A. oligospora* by modulating the transcription of sporulation-related genes.

Several studies have shown that PTPs participate in the regulation of fungal stress signaling by modulating the phosphorylation levels of MAPKs. In *S. cerevisiae*, Ptp2 and Ptp3 regulate the inactivation of MAPKs such as Hog1, Fus3, and Mpk1/Slt2, thereby participating in the negative feedback modulation of these pathways. Under heat stress conditions, Ptp2 and Ptp3 also prevent excessive activation of Hog1 and limit abnormal interaction between the HOG and CWI pathways, indicating that PTPs play a key role in maintaining MAPK signaling intensity and pathway specificity [24,25]. Here, AoPtp was found to play a key role in stress responses to oxidative-, osmotic-, and cell-wall-disturbing reagents, suggesting that AoPtp functions as a key regulatory factor in the perception and adaptation of *A. oligospora* to environmental changes. In addition, previous studies have demonstrated that AoPtp interacts with AoHog1 [32]. Importantly, we found that AoPtp interacts with AoFus3 and AoSlt2. Given the abnormal phenotypes observed in the Δ*Aoptp* mutant strains under osmotic, oxidative, and cell wall stress conditions, it can be hypothesized that AoPtp may contribute to the adaptation of *A. oligospora* to environmental alteration through its association with MAPK-related signaling pathways.

Trap formation in NT fungi is closely related to changes in nutritional status, environmental stimuli, and stress signals [30]. The Δ*Aoptp* mutants exhibited abnormalities in various stress responses and may exhibit altered stress signaling states or reduced activation thresholds. This altered stress response may contribute to the enhanced trap formation observed in the mutants. Interestingly, the response of the Δ*Aoptp* mutants to sorbitol stress was concentration-dependent. The mutants showed increased sensitivity to 0.25 M sorbitol but reduced sensitivity at 0.50 and 0.75 M. This non-monotonic response suggests that the loss of AoPtp may disrupt the fine regulation of osmotic stress signaling and lead to differential adaptive responses under distinct osmotic conditions. Considering that AoPtp interacts with AoHog1, deletion of AoPtp may alter the regulatory balance of the HOG-MAPK pathway by affecting Hog1-associated signaling dynamics. Under stronger osmotic stress, the absence of AoPtp may promote compensatory activation or prolonged activation of Hog1-related responses, thereby partially restoring osmotic adaptation and resulting in reduced sensitivity at higher sorbitol concentrations. Hog1 is a core component of the fungal HOG-MAPK pathway and is primarily involved in osmotic adaptation and stress responses [1,29]. In *A. oligospora*, Hog1 and Msb2 have been shown to participate in osmotic sensing, growth, trap formation, and spore production; Δ*hog1* and Δ*msb2* knockout mutants are highly sensitive to hyperosmotic conditions and exhibit reduced trap formation and nematocidal activity [1,32]. Therefore, the abnormal osmotic stress response of Δ*Aoptp* mutants may be associated with the activation or compensation of Hog1 signaling. Similarly, in *Fusarium graminearum*, FgPtp3 directly interacts with FgHog1 and inhibits the phosphorylation of FgHog1, thereby affecting HOG-MAPK signaling [40]. Therefore, different responses to the osmotic agent sorbitol observed in Δ*Aoptp* mutants of *A. oligospora* may be associated with altered Hog1 signaling.

In addition, Fus3 and Slt2 are closely associated with trap formation of *A. oligospora*. Recent studies have revealed that Fus3 is involved in mycelial growth, sporulation, autophagy, secondary metabolism, and trichome morphogenesis in *A. oligospora* [31]. Similarly, Slt2 and its upstream components, Bck1 and Mkk1, are involved in CWI, vegetative growth, trap formation, and the regulation of virulence; the absence of *Aobck1* and *Aomkk1* leads to severe cell wall damage, inability to form traps, and impaired nuclear development [41]. In this study, the Δ*Aoptp* mutants exhibited abnormal cell wall stress responses, abnormal trap morphology, and reduced number of nuclei. Furthermore, AoPtp was found to interact with AoFus3 and AoSlt2, suggesting that AoPtp may influence trap morphogenesis through Fus3/Slt2-related pathways, potentially involving cell wall remodeling, intercellular communication, and nuclear distribution.

Importantly, the increased trap production observed in Δ*Aoptp* mutants may be associated with the potential role of AoPtp as a negative regulator of MAPK signaling. PTPs generally regulate phosphorylation-dependent signaling cascades by reversing phosphorylation events and controlling the intensity and duration of MAPK activation [42]. Similar MAPK-associated tyrosine phosphatases have been reported to regulate fungal development, secondary metabolism, and pathogenicity in *A. flavus* [43]. Since AoPtp is a putative PTP and interacts with the MAPKs AoFus3 and AoSlt2, deletion of *Aoptp* may relieve the inhibitory regulation of these pathways, resulting in enhanced or prolonged Fus3/Slt2-related signaling. Previous studies in *A. oligospora* have demonstrated that MAPK-associated components, including Fus3, Slt2, Bck1, Mkk1, and Swi6, are essential for trap formation, and deletion of these components leads to reduced trap production or impaired trap development [28,31,41]. Therefore, the increased trap numbers observed in Δ*Aoptp* mutants may result from altered MAPK signaling dynamics caused by the loss of AoPtp-mediated negative regulation, although direct analysis of MAPK phosphorylation is required to confirm this hypothesis.

In *A. oligospora* and *A. flagrans*, trap formation requires the coordination of multiple processes, including hyphal polar growth, cell wall remodeling, localized swelling, and intercellular fusion [33,44]. In filamentous fungi, signal-mediated cell fusion is crucial for colony development. In *A. oligospora*, cell fusion-related proteins such as AoAdv-1 and AoSo are essential for hyphal cell fusion and trap formation. Their absence results in complete abolition of cell fusion, thereby preventing traps from closing to form normal fungal rings, accompanied by reduced spore production, abnormal stress responses, and LD accumulation [33,45]. Here, Δ*Aoptp* mutants were found to exhibit reduced cell fusion bridges and abnormal trap morphology; however, cell length exhibited no significant difference, suggesting that AoPtp primarily influences cell–cell recognition and fusion rather than hyphal longitudinal elongation. The Fus3 MAPK cascade in *A. oligospora* is critical for cell–cell communication, trap morphogenesis, and virulence [28]. The Slt2-class MAPKs are closely associated with CWI and cell wall remodeling [30]. Together with the confirmed interaction between AoPtp and AoFus3/AoSlt2, these findings suggest that AoPtp may participate in hyphal fusion and trap morphogenesis through modulating Fus3/Slt2 MAPK signaling.

LDs are important organelles involved in lipid storage, energy supply, and membrane lipid metabolism. The formation of phagosomes and the phagocytosis process require significant amounts of energy and membrane remodeling. LD size and volume undergo dynamic changes during phagosome formation and nematode phagocytosis [46]. In this study, the Δ*Aoptp* mutants exhibited markedly enlarged LDs and autophagic vacuoles, suggesting that AoPtp inactivation is associated with changes in lipid storage and energy-related processes. This shift may thereby affect the transition of *A. oligospora* from vegetative growth to phagocytic development. Previous studies have indicated that *A. oligospora* activates autophagy during the nematode-induced early stage, and that autophagy plays an indispensable function in mycelial growth, differentiation, and trap formation [36]. The Δ*Aoptp* mutants exhibited enlarged MDC-positive structures with reduced abundance, suggesting that AoPtp deficiency is associated with alterations in autophagy-related structures, including changes in their size and abundance.

In addition, AoPtp plays an essential role in metabolic process in *A. oligospora*, as its inactivation caused marked changes in secondary metabolite profiles. Recent studies have shown that several metabolites produced by NT fungi are closely associated with trap development, including 6-methyl-salicylic acid and arthrobotrisin [47]. Among these, arthrobotrisin has been shown to negatively regulate trap development in *A. oligospora* [47]. Here, loss of *Aoptp* resulted in increased trap numbers and a marked reduction in arthrobotrisin content. Accordingly, the transcription of genes belonging to the 215g gene cluster, which are involved in arthrobotrisin biosynthesis, was significantly reduced in Δ*Aoptp* mutants. These results suggest that altered arthrobotrisin biosynthesis may contribute to the enhanced trap formation observed in Δ*Aoptp* mutants.

Although domain analysis supports the AoPtp as a putative phosphatase and Y2H assays demonstrated its interactions with AoFus3 and AoSlt2, the catalytic activity of AoPtp and the direct dephosphorylation of these MAPKs were not examined in this study. Furthermore, BODIPY and MDC staining provide indirect evidence of changes in LD accumulation and autophagy-related structures but do not directly quantify total lipid content or autophagic flux. In addition, the ethyl acetate extraction method used for metabolomic analysis may also preferentially recover specific classes of extracellular secondary metabolites and may not fully represent the complete metabolome of *A. oligospora*. Therefore, further biochemical and cellular analyses will be required to clarify the underlying mechanisms.

## 5. Conclusions

In this study, we revealed the functional roles of AoPtp in a typical NT fungus. Our findings indicate that AoPtp contributes to vegetative growth, sporulation, and trap development, and is involved in autophagy-related processes and lipid metabolism. Inactivation of *Aoptp* caused a marked reduction in spore yield, cell fusion ability, and secondary metabolism, while simultaneously leading to a marked increase in trap formation and nematocidal activity. Furthermore, using a Y2H assay, we confirmed that AoPtp interacts with AoFus3 and AoSlt2, which suggests that AoPtp may participate in the regulation of spore production and trap formation through MAPK-related signaling pathways. Our findings reveal the functions of the PTP AoPtp in *A. oligospora*, and provide an important reference for further investigation of the AoPtp-associated MAPK regulatory network. Furthermore, this study establishes a robust foundation for probing the relationship between lifestyle transitions and pathogenicity in NT and other pathogenic fungi.

## Data Availability

All data generated or analyzed during this study are included in the published paper and associated supplemental files. RNA sequencing data were deposited in the National Microbiology Data Center under accession number NMDC10020819.

## Supplementary Materials

The following supporting information can be downloaded at https://www.mdpi.com/article/10.3390/jof12080595/s1. Figure S1: Phylogenetic analysis and validation of the Δ*Aoptp* mutant strains; Figure S2: Cell length of the WT strain and the Δ*Aoptp* mutant; Figure S3: Analysis of transcriptomic data of the Δ*Aoptp* mutant and WT strain; Table S1: Primers used for gene knockout and verification in this study; Table S2: Primers used for RT-qPCR analysis; Table S3: Primers used for yeast two-hybrid assay; Table S4: Predicted functional annotation of genes within the AOL_s00215g cluster associated with arthrobotrisin biosynthesis.
