# Supplementary material for: Protein Tyrosine Phosphatase Regulates Sporulation, Trap Morphogenesis, Stress Responses, and Secondary Metabolism in Arthrobotrys oligospora
Source: J Fungi (Basel). 2026 Aug 11;12(8):595. doi: 10.3390/jof12080595 (PMC13514423; doi:10.3390/jof12080595)
Supplement: Supplementary file 1 [file jof-12-00595-s001.zip › jof-4453621-supplementary.pdf]

Supporting Information

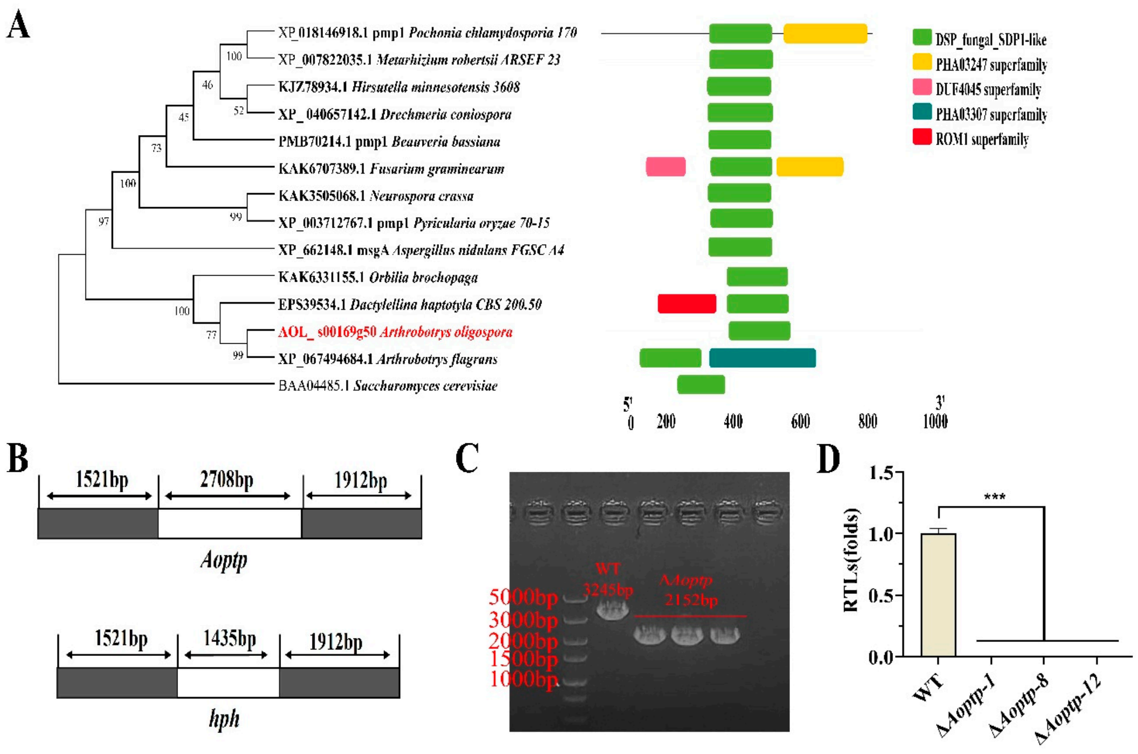

Figure S1. Phylogenetic analysis and validation of the Δ*Aoptp* mutant strains. (A) Phylogenetic tree of AoPtp and homologous proteins and conserved domains. (B) Schematic diagram of the gene knockout. (C) The transformants were verified using PCR amplification. (D) Validation of gene expression levels in the wild-type (WT) and Δ*Aoptp* mutant strains via RT-qPCR. Significant difference between the Δ*Aoptp* mutants and the WT strain are indicated with asterisks (Data represent mean ± SD, n = 3; Tukey's HSD test, *** $p < 0.001$).

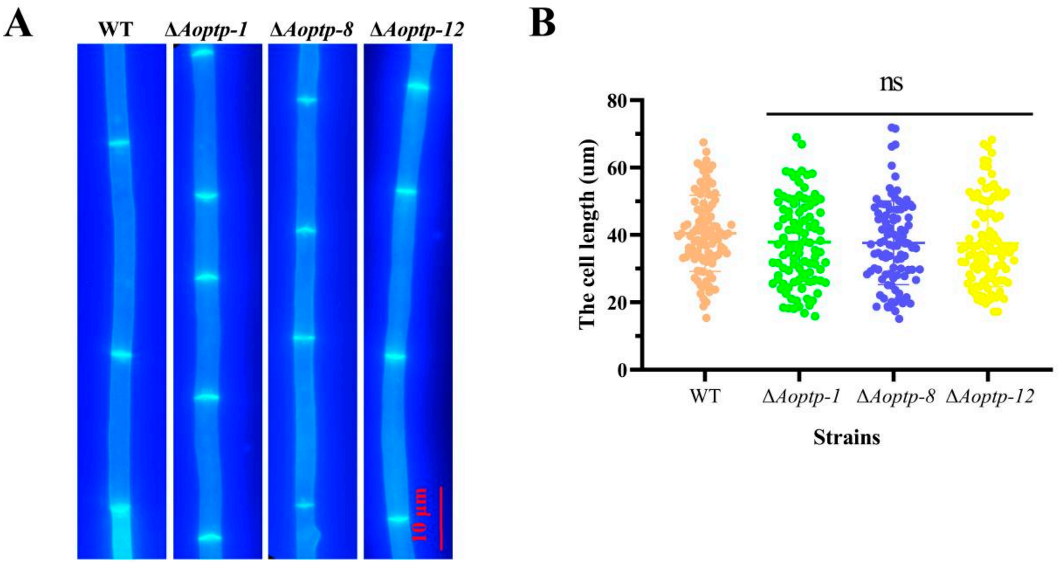

Figure S2. Cell length of the WT strain and the Δ*Aoptp* mutant. (A) Fluorescence images of the WT strain and the Δ*Aoptp* mutant (Bar = 10 μm) (B) Cell length statistics for the WT strain and the Δ*Aoptp* mutant. Significant differences between the Δ*Aoptp* mutants and the WT strain are indicated with asterisks (Data represent mean ± SD, n = 3; Tukey's HSD test, ns: no significant difference).

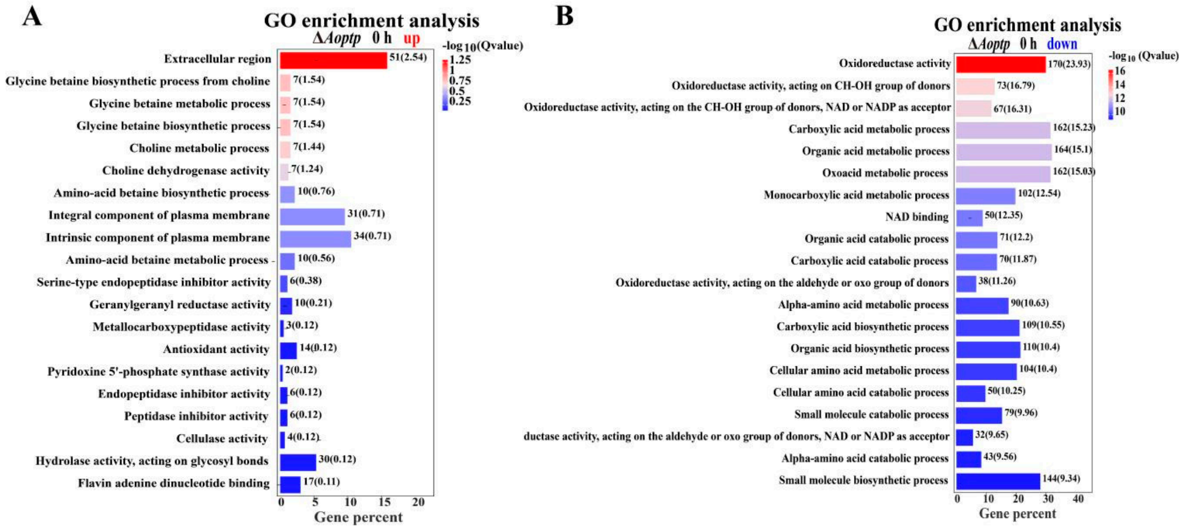

Figure S3. Analysis of transcriptomic data of the Δ*Aoptp* mutant and WT strain. (A, B) Top 20 GO enrichment analyses of upregulated and downregulated genes in the Δ*Aoptp* mutant at 0 h.

Table S1. Primers used for gene knockout and verification in this study.

	Sequence (5'-3')	Purpose
Aoptp-5F	GTAACGCCAGGGTTTTCCCAGTCACGACGAAGACGACAACGGCAAC A	Amplify the 5′ fragment
Aoptp-5R	ATCCACTTAACGTTACTGAAATCTCCAACGGTATGAACCAGTGGGAAG	
Aoptp-3F	CTCCTTCAATATCATCTTCTGTCTCCGACCACCGACTCCATCATTCA	Amplify the 3′ fragment
Aoptp-3R	GCGGATAACAATTTCACACAGGAAACAGCGTTGCGAGGTTACGACAG	
hph-5F	GTCGGAGACAGAAGATGATATTGAAGGAGC	Amplify the hph cassette
hph-5F	GTTGGAGATTTCAGTAACGTTAAGTGGAT	
YZ-5F	CACTGGTACTAGCGTCTCG	Transformants
YZ-3R	CTCCTCCTGTTGCTTCAC	verification via PCR
RT-5F	CCATGGGATCACAACACACCTA	Transformants
RT-3R	TAGGCTTCCTGTACCGTTTGTC	verification via RT-qPCR

Table S2. Primers used for RT-qPCR analysis

Sporulation-related genes	Sequence (5F)	Sequence (5F)
AOL_s00043g361 (*fluG*)	GATTCCAGTCCCGTGAATTC	GCTAAGGAGAGGATGGGCAT
AOL_s00054g811 (*velB*)	ATTCCGCAACTTCTCCCTCA	GGCATGTTTGGATTCTGGGG
AOL_s00080g63 (*abaA*)	AACTTTATGCGCCTTGTCGT	TTGGCTAGGTGGTCTGTACG
AOL_s00097g514 (*brlA*)	TTGAGGCCTCGATCCGTAGA	AGGTAGATGGCGCTGTTACG
AOL_s00173g221 (*wetA*)	TTACATGCCACCCCAAGTCC	CAATTGCAACTGCGTCCACA
AOL_s00169g18 (*veA*)	AAGCTACACCCAATCAACGC	TTGCGATGCTGACGATCTTG
AOL_s00007g157 (*flbC*)	CTCTCCGGCAAAGACAATC	GTCGACTGAGGATAGTAGCT
AOL_s00075g211 (*nsdD*)	ATTACGGCCGCCTAGTAGTC	CTCGTTTGGACCTGGTTGTG
AOL_s00006g570 (*hyp1*)	GCGGATCCAACATGAAGCTT	GGTTGACAACTGGGATGCTG

Table S3. Primers used for Yeast two-hybrid assay. The BD fusion protein was used as bait, and the AD fusion protein served as prey

	Sequence (5'-3')
AoLsg1-AD-F	gccatggaggccagtgaattcATGGGTCTACCAAAGGCCAAA
AoLsg1-AD-R	atgcccacccgggtggaattcATCATCAACAGTATTCGTACGTCTCTT
AoPkd1-AD-F	gccatggaggccagtgaattcATGGCGACGGCGGTTATG
AoPkd1-AD-R	atgcccacccgggtggaattcAATCGGAACTGCTAGTGCTGGA
AoSs1g-AD-F	gccatggaggccagtgaattcATGTCGACCGCACCTGTACC
AoSs1g-AD-R	atgcccacccgggtggaattcCTCAACTTCTAGGTATTTCAAAATCTTTT
AoCnaA-AD-F	gccatggaggccagtgaattcATGGCGGACGCAGCAGCT
AoCnaA-AD-R	atgcccacccgggtggaattcCATGCTGATCCTTCTGGCGA
AoPp1-AD-F	gccatggaggccagtgaattcATGTCGCTCAAGATGATATTTCTTG
AoPp1-AD-R	atgcccacccgggtggaattcAAACATGAAGAAAGCAAAAGCAAC
AoMid1-AD-F	gccatggaggccagtgaattcATGGCATTCAAGATATCTTGCCC
AoMid1-AD-R	atgcccacccgggtggaattcTCACAAAAGCCCCATAACCACC
AoFus3-AD-F	gccatggaggccagtgaattcATGTCTCGCCAGAACTCGAGC
AoFus3-AD-R	atgcccacccgggtggaattcCCGCATGATTTCTTCGAAGATC
AoHog1-AD-F	gccatggaggccagtgaattcATGGCAGAATTCATCCGCG
AoHog1-AD-R	atgcccacccgggtggaattcGAGTGGCGTATCGTTTTGGTCG
AoSlt2-AD-F	gccatggaggccagtgaattcATGGACTCCCTTCTGAACCGG
AoSlt2-AD-R	atgcccacccgggtggaattcATGGTACGCATGTCGACCATC
AoPtp-BD-F	atggccatggaggccgaattcATGGACGATCCGTTGATATTAAGA
AoPtp-BD-R	tcgacggatccccgggaattcTAGGACGTCGTAGATATTACGAATGG

Table S4. Predicted functional annotation of genes within the AOL_s00215g cluster associated with arthrobotrisin biosynthesis

Gene ID	Potential role in arthrobotrisin biosynthesis
AOL_s00215g273	Acetyltransferase
AOL_s00215g274	Short-chain dehydrogenase/reductase (SDR)
AOL_s00215g275	Regulatory protein
AOL_s00215g276	Prenyltransferase
AOL_s00215g277	Oxidoreductase
AOL_s00215g278	Cytochrome P450 monooxygenase
AOL_s00215g279	FAD-dependent monooxygenase
AOL_s00215g280	Cytochrome P450 monooxygenase
AOL_s00215g281	6-Methylsalicylic acid decarboxylase
AOL_s00215g282	Hydroxylase
AOL_s00215g283	Putative polyketide synthase (PKS)
